# Supplementary figures and images for: The human parasite, Toxoplasma gondii, is paralyzed without two components of the apical polar ring
Source: PLoS Pathog. 2026 Jun 26;22(6):e1014378. doi: 10.1371/journal.ppat.1014378 (PMC13387612; doi:10.1371/journal.ppat.1014378)

**Figure S1** Magenta-green version of the images in Fig 2C-E

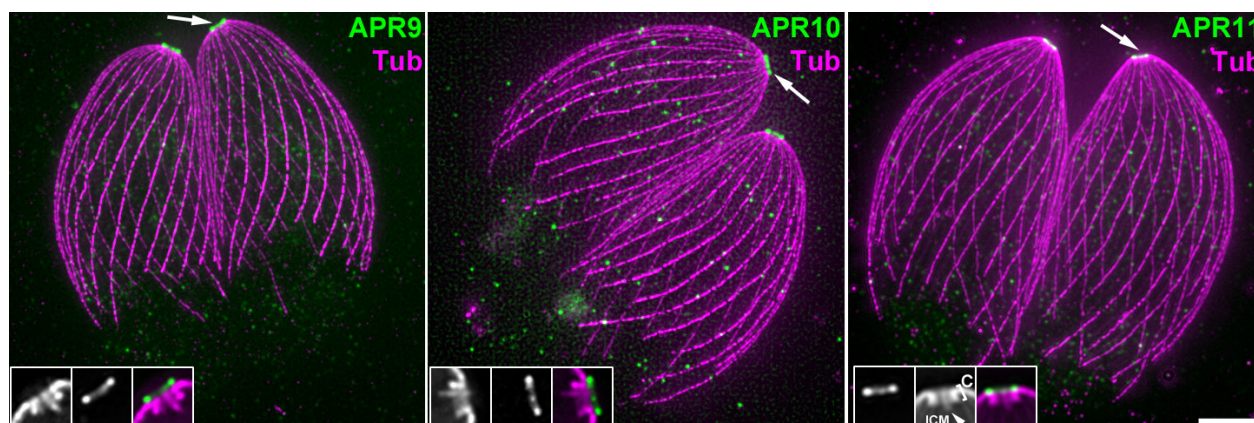

Supplement: S1 Fig — (PDF) [file ppat.1014378.s005.pdf]

**Figure S3** Magenta-green version of the images in Fig 3C-H

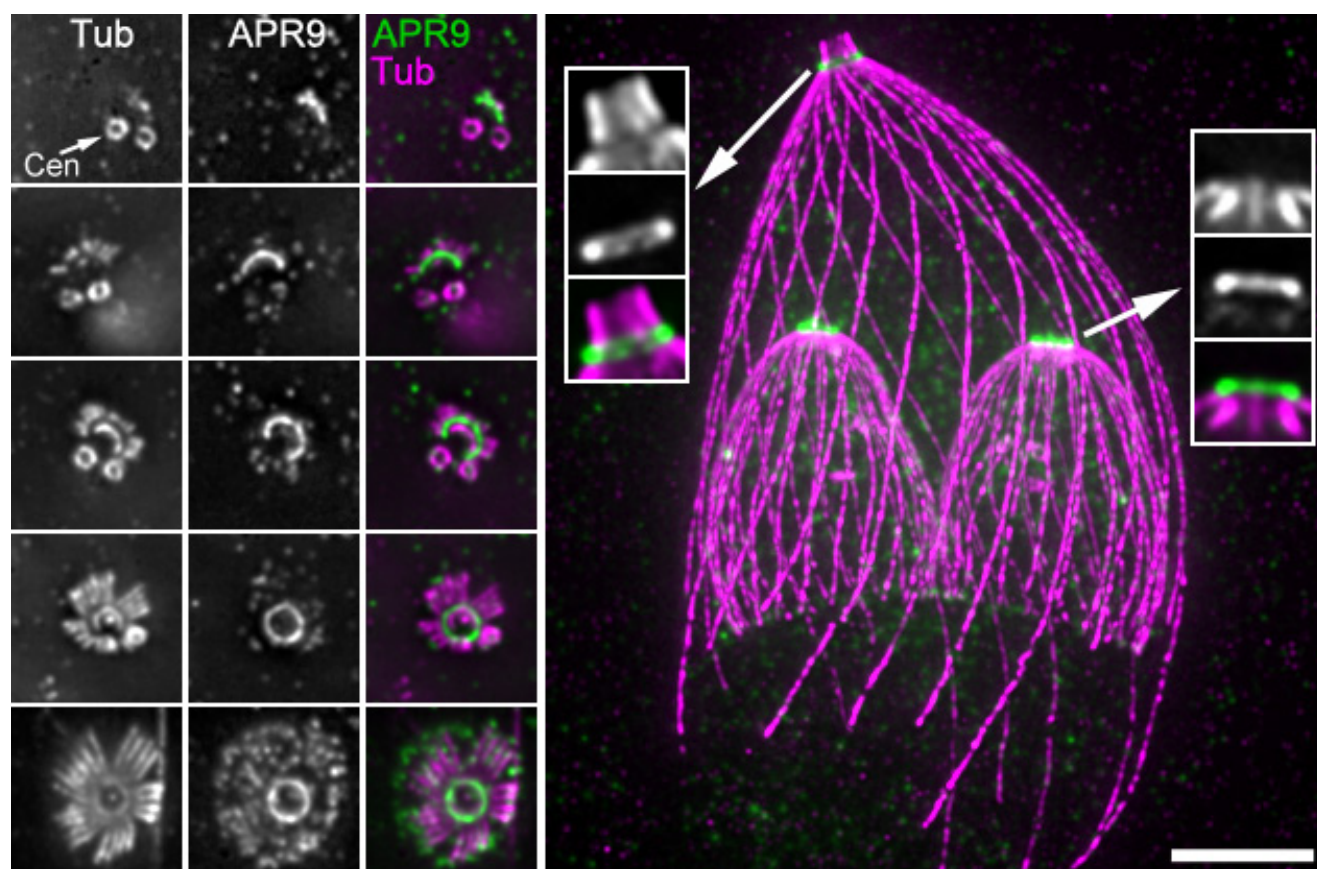

Supplement: S3 Fig — (PDF) [file ppat.1014378.s007.pdf]

**Figure S5** Magenta-green version of the images in Fig 4C-E

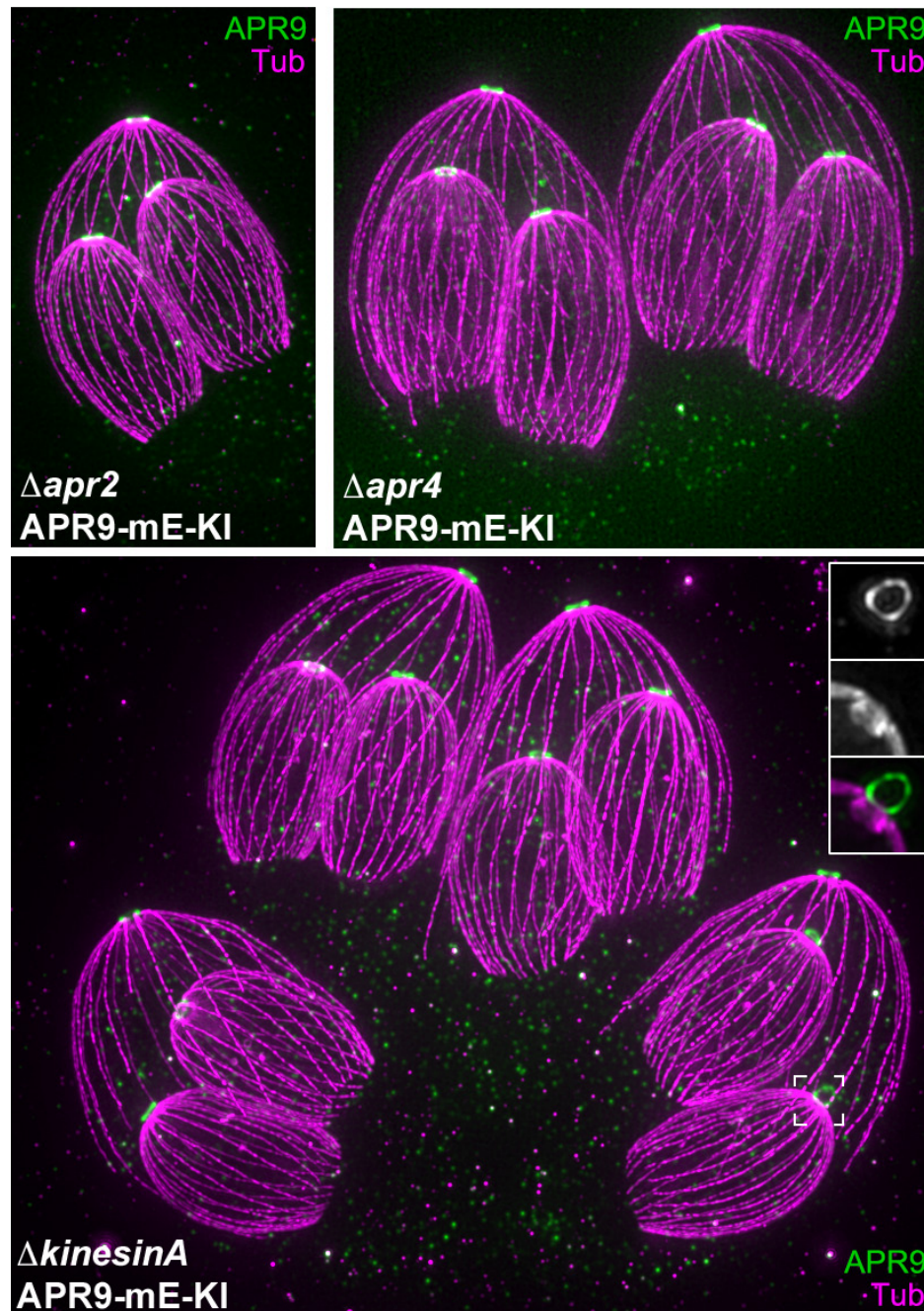

Supplement: S5 Fig — (PDF) [file ppat.1014378.s009.pdf]

**Figure S7** Magenta-green version of the images in Fig 8B

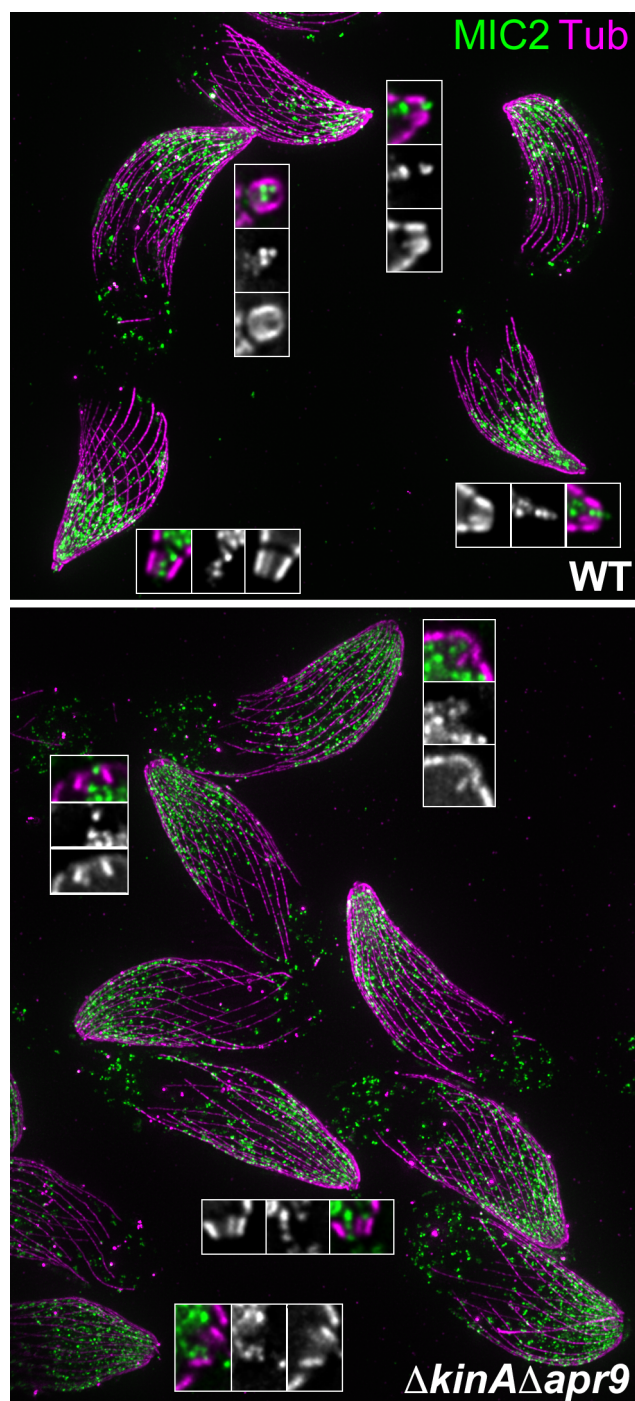

Supplement: S7 Fig — (PDF) [file ppat.1014378.s011.pdf]
