# Supplementary material for: The human parasite, Toxoplasma gondii, is paralyzed without two components of the apical polar ring
Source: PLoS Pathog. 2026 Jun 26;22(6):e1014378. doi: 10.1371/journal.ppat.1014378 (PMC13387612; doi:10.1371/journal.ppat.1014378)
Supplement: S2 Fig — The numbers of aligned residues were generated by TM-align (Version 20190822, Zhang, Y. and J. Skolnick, 2005. TM-align: a protein structure alignment algorithm based on the TM-score. Nucleic Acids Res 33, 2302–2309.). (PDF) [file ppat.1014378.s006.pdf]

Figure S2 Predicted structures of TgAPR9, TgAPR4, and orthologs from *Plasmodium berghei* and *Chromera velia* by AlphaFold3.

Toxoplasma gondii **APR4**
TGGT1_219500

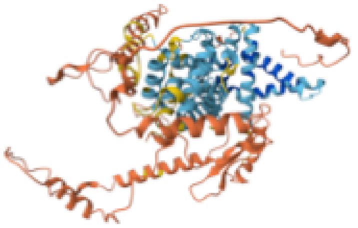

■ Very high (pLDDT > 90) ■ High (90 > pLDDT > 70)
■ Low (70 > pLDDT > 50) ■ Very low (pLDDT < 50)

Comparison	TM-score	Aligned residues
TgAPR4 vs TgAPR9	0.57	397
TgAPR9 vs PBANKA_091940	0.52	388
TgAPR9 vs Cvel_27126	0.16	236

Toxoplasma gondii **APR9**
TGGT1_223790

PBANKA_0919400.1

Cvel_27126

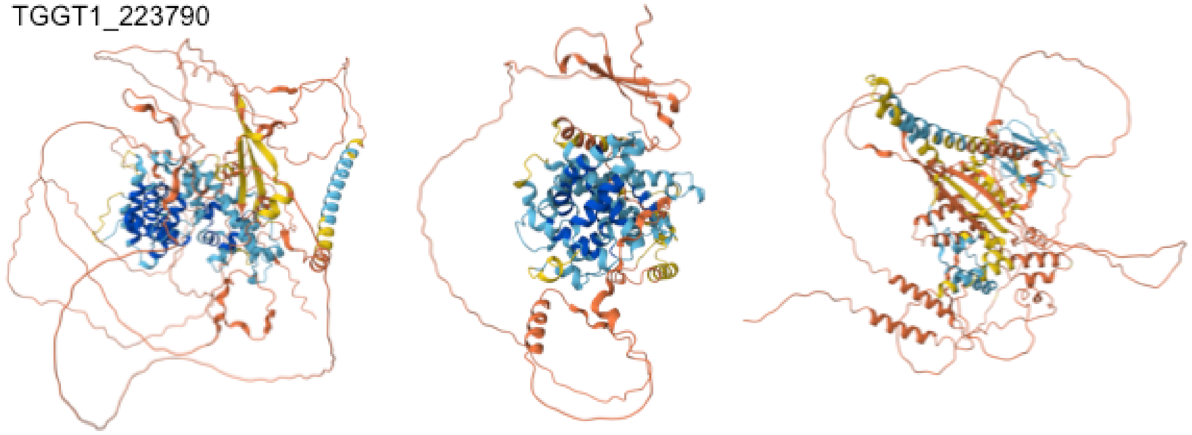

The numbers of aligned residues were generated by TM-align (Version 20190822, Zhang, Y. and J. Skolnick, 2005. TM-align: a protein structure alignment algorithm based on the TM-score. Nucleic Acids Res 33, 2302-2309.)
