# Supplementary material for: The human parasite, Toxoplasma gondii, is paralyzed without two components of the apical polar ring
Source: PLoS Pathog. 2026 Jun 26;22(6):e1014378. doi: 10.1371/journal.ppat.1014378 (PMC13387612; doi:10.1371/journal.ppat.1014378)
Supplement: S4 Fig — A. Schematic for the predicted APR4 locus in WT, mE- tagged APR4 knock-in, and Δapr4 lines. Restriction sites, hybridization targets of the Southern blot probes for the apr4 coding region (CDS probe, orange bar), regions upstream (“5’ UTR probe”, blue bar) and downstream (“3’ UTR probe”, purple bar) of the CDS, and the corresponding DNA fragment sizes expected are indicated. B. Southern blots confirmed the homologous integration of the mE-APR4 fusion in the knock-in line, and the deletion of the apr4 locus in the Δapr4 lines. (PDF) [file ppat.1014378.s008.pdf]

Figure S4

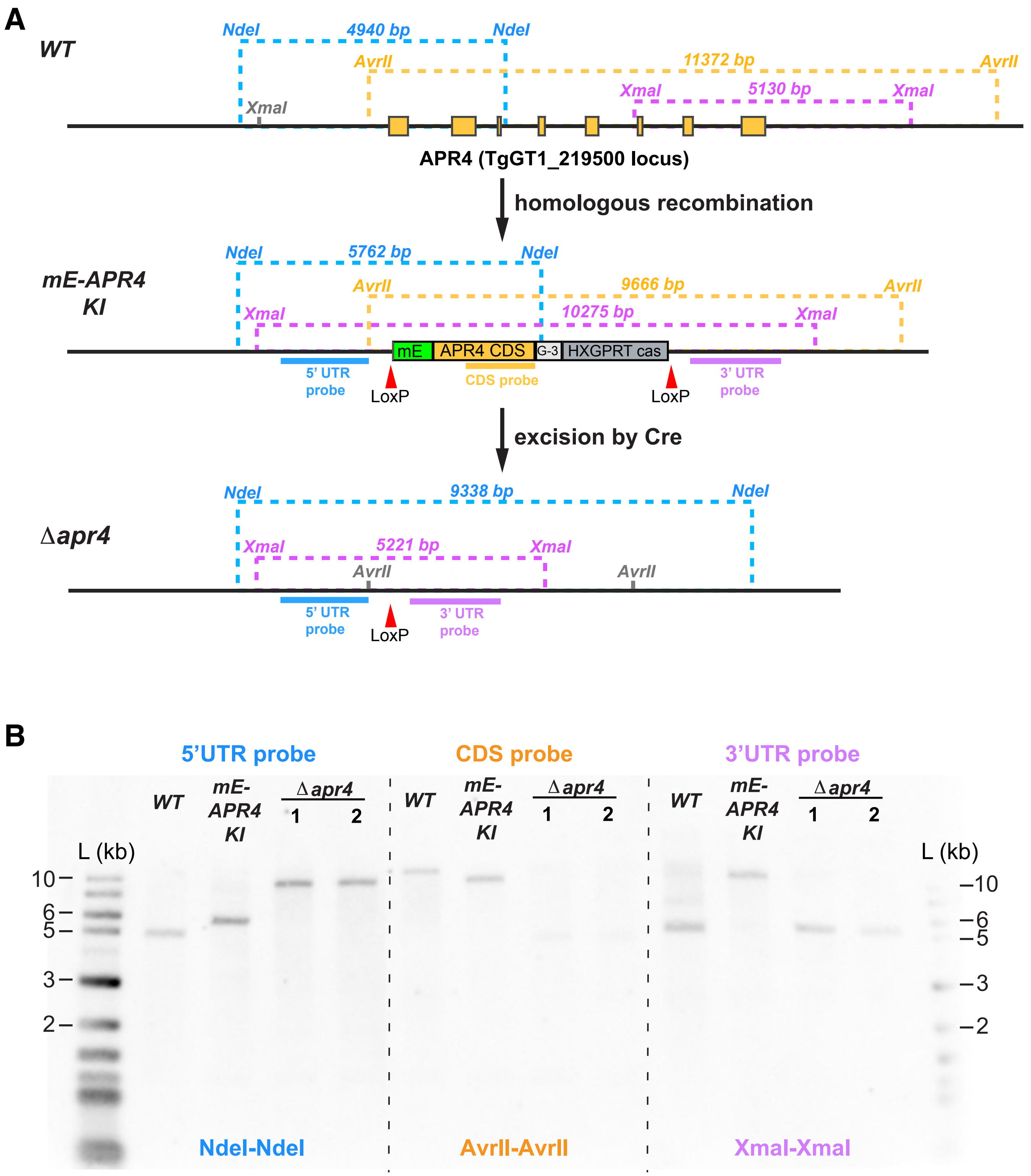

Fig. S4 Southern blot analysis of the *apr4* locus in the WT, *mEmeraldFP-APR4* (mE-APR4 KI) and Δ*apr4* parasites.

A. Schematic for the predicted APR4 locus in WT, *mE-* tagged APR4 knock-in, and Δ*apr4* lines. Restriction sites, hybridization targets of the Southern blot probes for the *apr4* coding region (CDS probe, orange bar), regions upstream ("5' UTR probe", blue bar) and downstream ("3' UTR probe", purple bar) of the CDS, and the corresponding DNA fragment sizes expected are indicated.

B. Southern blots confirmed the homologous integration of the mE-APR4 fusion in the knock-in line, and the deletion of the *apr4* locus in the *Δapr4* lines.
