# Supplementary material for: The human parasite, Toxoplasma gondii, is paralyzed without two components of the apical polar ring
Source: PLoS Pathog. 2026 Jun 26;22(6):e1014378. doi: 10.1371/journal.ppat.1014378 (PMC13387612; doi:10.1371/journal.ppat.1014378)
Supplement: S6 Fig — (PDF) [file ppat.1014378.s010.pdf]

Figure S6

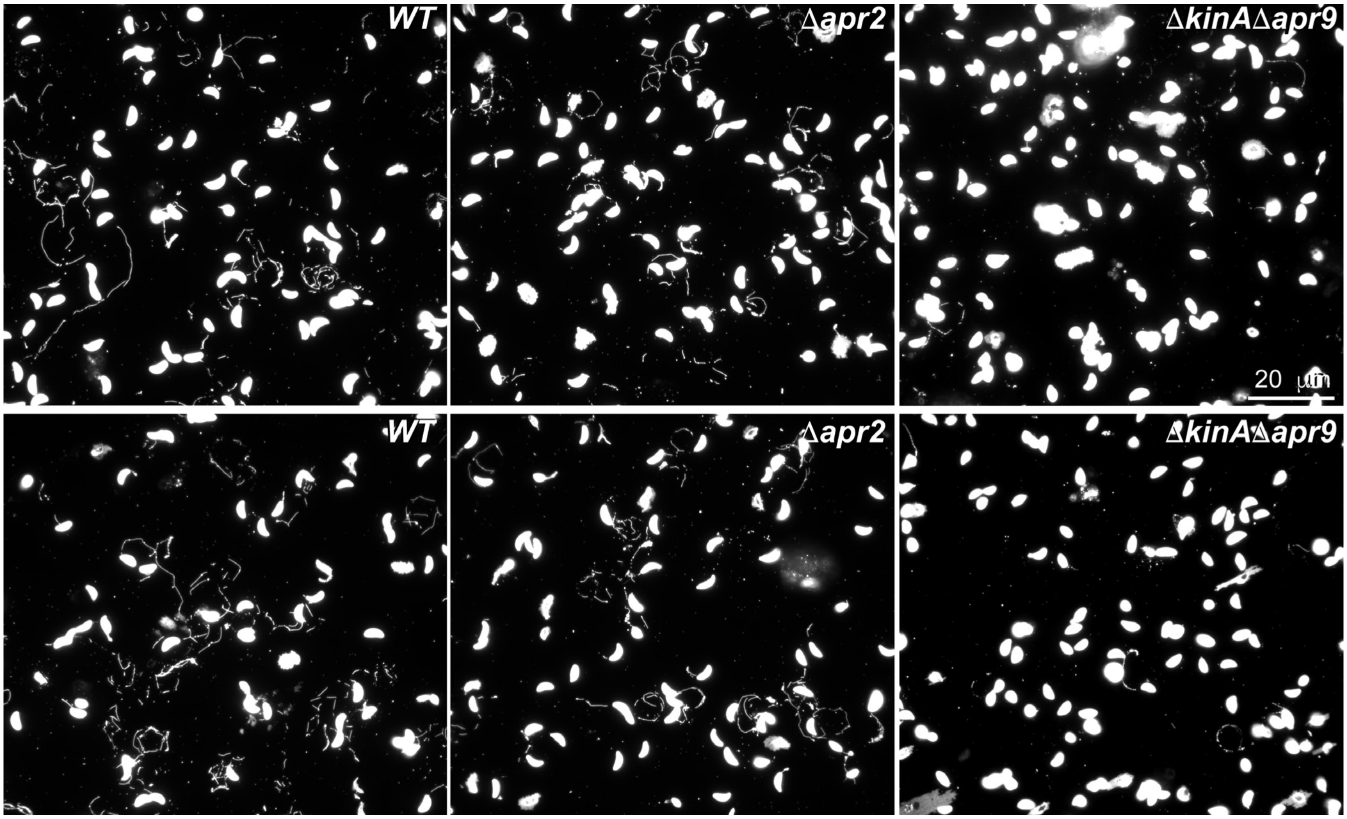

Fig. S6. Two additional sets of images of 2-D gliding motility assay of RHΔku80 parental (WT), Δapr2, and ΔkinesinAΔapr9 parasites.
