# Supplementary material for: The human parasite, Toxoplasma gondii, is paralyzed without two components of the apical polar ring
Source: PLoS Pathog. 2026 Jun 26;22(6):e1014378. doi: 10.1371/journal.ppat.1014378 (PMC13387612; doi:10.1371/journal.ppat.1014378)
Supplement: S2 Table — Values represent background-subtracted intensities of the secreted MIC2 band in the A23187-induced samples relative to WT parasites in DMEM growth medium, normalized to the corresponding tubulin loading control in the pellet fraction. (PDF) [file ppat.1014378.s013.pdf]

Table S2. Quantification of A23187-induced MIC2 secretion of WT and *ΔkinesinAΔapr9* parasites in DMEM growth medium and L15 imaging medium. Values represent background-subtracted intensities of the secreted MIC2 band in the A23187-induced samples relative to WT parasites in the DMEM growth medium, normalized to the corresponding tubulin loading control in the pellet fraction.

	Media (+A23187)	replicate 1	replicate 2	replicate 3	Average ±SEM
RHΔku80 (WT)	DMEM	100%	100%	100%	100%
	L15	172%	224%	77%	158±43%
ΔkinesinA Δapr9	DMEM	12%	18%	12%	14±2%
	L15	22%	39%	23%	28±6%
