# Supplementary material for: The human parasite, Toxoplasma gondii, is paralyzed without two components of the apical polar ring
Source: PLoS Pathog. 2026 Jun 26;22(6):e1014378. doi: 10.1371/journal.ppat.1014378 (PMC13387612; doi:10.1371/journal.ppat.1014378)
Supplement: Attachment — Submitted filename: Response_to_reviewers_RV1.pdf [file ppat.1014378.s017.pdf]

In the following Response to the reviews, the original words of the reviewers are in *italics*, and our responses in plain text. Text from our revised manuscript is included in " ".

Please note that, when examining the figures, please use the links in the manuscript PDF to download the original figure files, as the quality of the figures in the PDF converted by the PLoS web server is poor due to overcompression. At the resubmission stage, we have no choice but to use the PDF conversion at PLoS.

Major comments:

Reviewer #1:

1. *Line 90. It is unclear whether APR4 or APR9 is the closest homolog to the proteins referenced in Figure 3. While this does not alter the main conclusions, inclusion of a phylogenetic tree encompassing all relevant homologs would clarify the relationship between APR4, APR9, and related proteins. This would help establish which T. gondii APR protein is most similar to those identified in other apicomplexans.*

A phylogenetic tree of APR4/9 homologs is now included as the new Figure 3B.

Line 189 / Figure 5D–F. While the authors likely distinguish apical and basal ends readily, this distinction is not obvious to the reader. In particular, the WT and double mutant images are difficult to interpret based on cell shape alone. A supplemental movie showing actin accumulation and directionality of movement would greatly improve clarity and should be feasible to provide.

Supplemental movies corresponding to these panels (new Figure 6A-C) are now included as Videos S2-S4,

Figure 6E. Given the reduced secretion observed in the mutant, are there any differences in MIC2 labeling between WT and the double mutant following A23187 treatment? This analysis could be informative.

Expansion microscopy images of MIC2 labeling of the WT and *ΔkinesinAΔapr9* parasites after A23187 treatment are now included as Figure 8B. The data revealed interesting information regarding MIC2 containing vesicles within the conoid. However, it does not provide an obvious explanation of the MIC2 secretion defect of the *ΔkinesinAΔapr9* parasite.

P7, line 253: "Interestingly, in extracellular parasites treated with A23187, MIC2-containing vesicles are found within the extended conoid of the WT parasites, as well as within the retracted and partially extended conoid in the Δ*kinesinA*Δ*apr9* parasites (**Fig 8B, Fig S7**)."

Reviewer #2:

1. The motility defect of the double mutant is not entirely clear to me. The authors assessed motility by videomicroscopy after A23187 induction. Based on Figure 5B and Video S1, it is unclear whether the mutant exhibits sporadic movement or active motility within the vacuole.

Most of the *ΔkinesinAΔapr9* parasites are immobile after treatment with A23187. Occasionally, one or two parasites in a vacuole display sporadic movement.

For the APR2 mutant, although parasites egress normally from the vacuole, no gliding motility is visible post-egress, in contrast to wild-type parasites which exhibit clear gliding motion in Video S1. Performing a classical gliding motility assay (e.g., detection of SAG1 trails) in both the APR2 and APR9/KinA mutants, along with assessing parasite dispersion after egress by IFA, would further strengthen the authors' conclusions. This point may warrant attention, as some of the authors' conclusions (lines 193-196 ; 218-219 ; 278-280 and 284-286) rely on the assumption that the APR2 parasite is not defective in gliding motility. However, a previous study reported that the APR2 mutant displays normal egress upon BIPPO-induced stimulation but shows impaired post-egress dispersion, consistent with a motility defect, and also produces fewer trails (Ren et al. PNAS 2024). If APR2 is truly motile under these conditions, this would challenge the current model linking conoid extrusion and actin flux dynamics to parasite motility.

a. It is not clear to us why the reviewer concluded that "*For the APR2 mutant.... no gliding motility is visible post-egress*", since there was no video for the Δ*apr2* parasite included in our last submission. However, we have now included a live egress movie of the Δ*apr2* parasite (Video S3), which shows that the Δ*apr2* parasites not only actively egress from the vacuole, but also continue to move actively by gliding motility after egress.

b. We have now also performed 2-D gliding motility assays. The results are included as New Figure 6D and Supplementary Figure S6. We found that the difference in trail density between the wild-type and Δ*apr2* parasite is not pronounced. However, while long, non-circular trails were readily detectable in the WT samples, the longer trails deposited by the Δ*apr2* parasite tended to be circular in nature. This might account for the modest invasion defect of the Δ*apr2* parasite we observed previously (Arias Padila and Munera Lopez et al. 2024 Journal of Science). The difference between the *ΔkinesinAΔapr9* parasite and the other two lines was much more striking. The trail density of the *ΔkinesinAΔapr9* parasite was much lower than those of both WT and Δ*apr2* parasites. In other words, even though the *Δapr2* and *ΔkinesinAΔapr9* parasites both accumulate an apical actin cap and are defective in conoid extension upon A23187 treatment, their motile behaviors during egress and on a 2-D surface are qualitatively different.

P6, line 211: "To examine the parasite motile behavior in a host-cell-free environment, we carried out two-dimensional (2-D) gliding motility assay of WT, Δ*apr2*, and Δ*kinesinA*Δ*apr9* parasites (**Fig 6D**, **Fig S6)**. We found that while long, non-circular trails were readily detectable in the WT samples (**Fig 6D**, yellow arrows), the longer trails deposited by the Δ*apr2* parasite tended to be circular in nature (yellow arrowheads). This might account for the modest invasion defect of the *Δapr2* parasite [28]. The difference between the *ΔkinesinAΔapr9* parasite and the other two lines was much more striking. Very few trails were detected in the *ΔkinesinAΔapr9* parasite samples. Therefore even though both the Δ*apr2* and *ΔkinesinAΔapr9* parasites accumulate an apical actin cap upon A23187 treatment, their motile behaviors during egress and on a 2-D surface are qualitatively different."

c. We performed IFA based egress assays prior to the first submission of the manuscript. We found that because of the large number of uninvaded *ΔkinesinAΔapr9* parasites, which cannot be distinguished from freshly egressed parasites in this assay, it is difficult to accurately assess egress efficiency for this line by imaging fixed parasites. This was why we opted for assessing egress using live cell imaging.

Reviewer #3: *My main criticism of the manuscript in its current form is why the KinesinA/APR9 dual knockout, among all the other potential dual-KOs, was selected for analysis. Excluding APR4/APR9 due a lack of a strong phenotype is reasonable, but I'm curious about what other duals were generated, and what rationale was used to select the KinesinA dual KO out of the other possibilities. Specifically, it seems that APR2 would make good sense as second knockout in conjunction with APR9, considering that it was the bait in the initial IP. While APR9 localization does not appear to change in the APR2 knockout, there seems to be some connection between the two proteins that merits further study. For example, a stronger impact on conoid extrusion and motility in the APR2/APR9 dual knockout could suggest that they are working along the same pathways. More background on APR2 in the introduction and the discussion would also be useful.*

We decided to focus on the *ΔkinesinAΔapr9* parasite because its phenotype is much more severe than all the other mutants generated during this study. We did generate a APR2-APR9 double knockout mutant (*Δapr2Δapr9*), which has a modest defect in the lytic cycle. We did not include it in the original manuscript to streamline the narrative. We have now included the plaque assay and Southern blot results for the *Δapr2Δapr9* parasite as part of Figure 4, and revised the Results and Discussion accordingly.

Results, p5, line 139: "Because APR4 is a close homolog of APR9, we generated a double-knockout line (*Δapr4Δapr9*) by transiently expressing the Cre-recombinase in the *Δapr4:mE-APR9* knock-in parasite to excise the *mE-APR9 knock-in* locus (Fig 4A-B). We similarly generated two additional double knockout lines: a *Δapr2Δapr9* line, because APR9 was identified in the APR2 IP, and a *ΔkinesinAΔapr9* line, because KinesinA shows a synergistic effect with two other apical polar components, APR1 and APR2 [17, 28]. "

Discussion, p8, line 291: "However, in *Toxoplasma,* the Δ*apr9,* Δ*apr4,* Δ*apr4*Δ*apr9* and Δ*apr2*Δ*apr9* parasites all have only modest defects in the lytic cycle (this work and [29])."

We have also now included some more background on APR2 in Introduction:
P3, line 55: "The removal of APR2 has only a minor impact on MT organization [28]. However, due to its early recruitment to the apical polar ring, we decided to use it as the bait in immunoprecipitation."

Other relevant background information of APR2 (e.g. its impact on actin cap accumulation) was described in the Results when comparing the phenotypes of various mutants.

P6, line 204:"Previously it was shown that the knockdown of APR2 results in apical mE-actin-Cb concentration when treated with BIPPO, an inhibitor of apicomplexan phosphodiesterases implicated in the Ca^{2+} signaling pathways [29, 50]. We also observed the formation of a prominent apical mE-actin-Cb concentration when our APR2 knockout line (Δ*apr2*) was treated with A23187 (**Fig 6B)**."

Minor comments:

Reviewer #1:

1. *Line 50. In addition to the authors' prior work (ref. 26), a recent study (Engelberg, 2025, mBio) demonstrates that γ-tubulin transiently associates with the APR to nucleate microtubules. This finding further supports the authors' assertion regarding the APR's role in organizing cortical microtubules and should be cited.*

Thanks to the reviewer for pointing this out. The Engelberg paper is now cited in the Introduction along with the Haase paper (2024, MBoC) that reported similar findings.

P3, line 51:"Two other studies also showed that the MT nucleator γ-tubulin associates with the apical polar ring transiently during the early stage of daughter assembly, further supporting the role of the apical polar ring in initiating the new MT array [33, 34]"

2. *Line 78. The authors state that three new candidates were identified. While APR9–11 are clearly the most abundant uncharacterized proteins in the GFP-trap experiment (Table S1), several additional uncharacterized proteins (e.g., TGGT1_218720, TGGT1_275670, TGGT1_271880, TGGT1_229920) appear at levels comparable to known APR components such as APR1, APR5, and APR7. Were these proteins tested for APR localization and excluded, or were only the top hits prioritized? Clarification would be helpful.*

TGGT1_218720, TGGT1_275670, TGGT1_271880, TGGT1_229920 have all been characterized in other studies. TGGT1_218720 (CDPK6) and TGGT1_275670 (IMC15) are localized to the parasite apex and the basal complex. TGGT1_271880 is Pcr7, a preconoidal ring protein. TGGT1_229920 is the cytochrome c oxidase subunit ApiCOX35. The text in Results has been revised to include this information. These genes are also more prominently labeled in Table S1.

P4, line 81:"Known proteins among the top 15 hits include APR2, seven other components of the apical polar ring (KinesinA, APR1, APR3-5, APR7, MLC3), a preconoidal ring protein (Pcr7), two proteins previously localized to the parasite apex and other cytoskeletal structures (CDPK6 and IMC15), and the cytochrome c oxidase subunit ApiCOX35 [20, 27, 29, 38-41](**Table S1, Fig 1C**)."

3. *Line 110. "mE" is defined only on line 112 but could be introduced earlier (e.g., line 81) when mEmerald knock-ins are first mentioned.*

"mE" is now introduced when mEmerald knock-ins are first mentioned (line 77).

4. *Lines 130 and 263. The manuscript refers to broad conservation of APR9; however, the relationship between APR4 and APR9 homologs remains unresolved. It may be more accurate to refer to APR4/9 when discussing conservation, as done on line 93.*

Because these sections lead by discussing APR9 specifically, we feel that it reads better as is. "APR4/9 homologs" are used in other places in the manuscript when discussing this protein family (lines 100, 290, 590).

5. *Figure 2. The ordering of images in panel A makes the figure harder to follow. It would be more intuitive to present APR9 (223790) between APR11 and APR10, or simply in numerical order (APR9, APR10, APR11), consistent with Figure 1C. In panel B, the first lane is not labeled; when printed, it appears as a single band (~1.5 kb), making it difficult to interpret. It is only clear upon closer inspection that this is a ladder. Labeling the lane explicitly and/or adjusting contrast or cropping the gel to emphasize marker labels would improve clarity and consistency between the two gels.*

This figure has now been reorganized in the order of APR9, 10 and 11. The DNA gels are cropped such that the genomic PCR result for each locus is aligned with the corresponding diagram.

6. *Figure 6B. Three images are labeled as "retracted conoid" for the double mutant. It appears the authors intended to illustrate a range of phenotypes; however, panel 6D introduces a "partially extended" category that is not clearly represented in 6B. If any of the images correspond to this intermediate phenotype, they should be labeled accordingly.*

Thanks to the reviewer for the suggestion. One of the *ΔkinesinAΔapr9* EM images is now relabeled as "partially extended".

7. *Line 269. Is the P. berghei ortholog specific to APR9, or does it represent an APR4/9 homolog? Clarification would be useful.*

A phylogenetic tree of APR4/9 homologs is now included as the new Figure 3B. The *P. berghei* ortholog is homologous to both APR9 and APR4.

P8, line 290: "The *P. berghei* ortholog of APR4/9 is predicted to be highly fitness-conferring (denoted "essential" in [56] and *PlasmoDB*)."

Reviewer #2:

1. *One intriguing observation is the effect of APR9/KinA depletion on MIC2 secretion, which cannot be attributed to SPMT disorganization, as these structures appear largely normal. It would be helpful if the authors could at least speculate on how microneme release might be compromised in this mutant.*

We have now included a speculation on how the apical polar ring might be involved in microneme secretion.

P8, line 326: "Ultrastructural studies show that the apical complex is structurally coupled to secretory organelles [8, 23, 58, 59]. The mechanics of micronemal secretion, however, remains elusive. The apical polar ring might facilitate protein release by interacting with surface structures on micronemal vesicles. Further studies that explore if and how KinesinA and functionally connected proteins such as APR1 and APR9 interact with micronemal vesicles to control secretion will provide useful insights."

2. *The schematic in Figure 1A shows two PCR rings, whereas recent data indicate the presence of three.*

Figure 1A has now been revised to show three preconoidal rings.

3. *In the introduction, line 50: "This observation supports the hypothesis that the apical polar ring initiates and templates new MT arrays…" A recent study (Li et al., Nature Communications, 2025) showed that in the absence of APR, SPMTs are initiated but not organized. This supports the view that the APR is not required for SPMT initiation but rather for their proper organization. Please revise the sentence accordingly.*

Li et al. (Nature Communications, 2025) showed that the loss of ASAF1, an early apical polar ring component, resulted in failed daughter assembly. The paper proposes that ASAF1 is important for the assembly of the cytoskeletal apical complex (a.k.a. the conoid complex). It examined a known apical polar ring component, RNG2, which was found to form discrete clusters upon ASAF1 depletion. This is a striking and important study. However, our interpretation of certain aspects of the work is different from that of the reviewer's. Firstly, because daughter formation is highly perturbed, it is difficult to assess whether the cytoplasmic microtubules in the ASAF1 mutant have the same origin as the cortical MTs/SPMTs. Secondly, the complete ablation of the apical polar ring upon ASAF1 depletion has yet to be proven. The apical polar ring is a multi-layered structure that contains more than a dozen known components. Examining only one component is not sufficient to conclude that the entire apical polar ring structure is abolished. For example, in our own work, we found that in the *ΔkinesinAΔapr1* mutant, RNG1-labeling is fragmented, but APR2 still forms an intact ring (Fig 6, Arias Padila and Munera Lopez et al. 2024 Journal of Science).

4. *In Figure 4G, the p-values of 0.01 and 0.04 are not clearly assigned. I assume they correspond to complemented clones 1 and 2.*

The bar graph has been revised to more clearly indicate that the p-value of 0.01 corresponds to *ΔkinA*:mE-APR9 KI and 0.04 corresponds to complemented clone 1.

5. *A graph showing the invasion defect and standard deviation would be easier to read than a table.*

We agree with the reviewer that a bar graph is more visual. However, a table makes it possible to include the matrix of the p-values, which is challenging to display in a graph.

6. *Figure 5C: The p-values are missing from the graph.*

We have now added p-values in the description of this panel (new Figure 5B) in the main text.

P6, Line 178: "Compared with the WT, *Δapr9, ΔkinesinA,* and the complemented lines, it takes significantly longer for the first *ΔkinesinAΔapr9* parasite to become mobile and initiate active egress, with a p-value of 0.0003, 0.002, 0.0005, and 0.0003, respectively. Furthermore, for the WT, *Δapr9, ΔkinesinA,* and complemented lines, the median time for ≥ 50% of the parasites in a vacuole to actively egress is around 50 seconds. In contrast, the *ΔkinesinAΔapr9* line did not reach this threshold in any vacuole during the 300-second observation period (p < 0.0001)."

Reviewer #3:

1. *I would recommend using vision-deficiency-compatible channels for the light microscopy images, such as magenta-green.*

Magenta-green versions of the dual-color fluorescence images in Figures 2, 3, 4 and 8 are now included as Supplemental Figures S1, S3, S5, and S7.

2. *Fig 5, Fig6- I think the graphs are already quite small in a full-page layout and would not work well in a conventional figure layout. The graph text is already difficult to read.*

Thanks to the reviewer for pointing this out. With the inclusion of the new data, the original Figure 5 is now split into new Figures 5 and 6. The original Figure 6 is now split into new Figures 7 and 8. The graphs are enlarged to make the text more readable.

3. *Fig 6- the insets in A could be a lot clearer, it's hard to understand what you are looking at. I would perhaps inset the microtubule issue shown with the arrow as well as the apical connection issue, and make them both significantly larger.*

This panel (now Figure 7A) and the corresponding figure legend have now been revised. The inset including the parasite apex is further enlarged to 2.5X. For the two detached cortical MTs further down at the parasite body, a projection of a substack close to the parasite surface is included as an inset (1X) to provide more clarity.

Figure 7A legend (p15, line 660): "The left inset (1X) is a projection of a substack close to the parasite surface and includes two detached cortical MTs. The right inset (2.5X) is a projection of a substack that includes the parasite apex, with the arrowhead indicating the gap between the cortical MTs and the conoid ("C")."
