# Supplementary material for: The human parasite, Toxoplasma gondii, is paralyzed without two components of the apical polar ring
Source: PLoS Pathog. 2026 Jun 26;22(6):e1014378. doi: 10.1371/journal.ppat.1014378 (PMC13387612; doi:10.1371/journal.ppat.1014378)
Supplement: Peer review history

# Peer review: Decision Letter 0

**DOI:** 10.1371/journal.ppat.1014378.r001

**Authors:** Margaret Phillips, Michael Reese

13 Apr 2026

PPATHOGENS-D-26-00608

The human parasite, Toxoplasma gondii, is paralyzed without two components of the apical polar ring

PLOS Pathogens

Dear Dr. Hu,

Thank you for submitting your manuscript to PLOS Pathogens. After careful consideration, we feel that it has merit but does not fully meet PLOS Pathogens's publication criteria as it currently stands. Therefore, we invite you to submit a revised version of the manuscript that addresses the points raised during the review process.

Please submit your revised manuscript by Jun 12 2026 11:59PM. If you will need more time than this to complete your revisions, please reply to this message or contact the journal office at plospathogens@plos.org. When you're ready to submit your revision, log on to https://www.editorialmanager.com/ppathogens/ and select the 'Submissions Needing Revision' folder to locate your manuscript file.

Please include the following items when submitting your revised manuscript:

* A letter that responds to each point raised by the editor and reviewer(s). You should upload this letter as a separate file labeled 'Response to Reviewers'. This file does not need to include responses to any formatting updates and technical items listed in the 'Journal Requirements' section below.

* A marked-up copy of your manuscript that highlights changes made to the original version. You should upload this as a separate file labeled 'Revised Manuscript with Track Changes'.

* An unmarked version of your revised paper without tracked changes. You should upload this as a separate file labeled 'Manuscript'.

If you would like to make changes to your financial disclosure, competing interests statement, or data availability statement, please make these updates within the submission form at the time of resubmission. Guidelines for resubmitting your figure files are available below the reviewer comments at the end of this letter.

We look forward to receiving your revised manuscript.

Kind regards,

Michael L Reese, PhD

Academic Editor

PLOS Pathogens

Margaret Phillips

Section Editor

PLOS Pathogens Sumita Bhaduri-McIntosh

Editor-in-Chief

PLOS Pathogens

orcid.org/0000-0003-2946-9497

Michael Malim

Editor-in-Chief

PLOS Pathogens

orcid.org/0000-0002-7699-2064

**Additional Editor Comments:**

Thank you for your submission to PLoS Pathogens. As you will see, all three reviewers thought the manuscript was of high quality, but have suggestions on how to improve the manuscript. Please respond productively to all criticism in your resubmission. I note that Reviewer number 3 suggests including data for an APR2/9 double KO may enhance the manuscript. While I agree that this may be potentially interesting, I do not think it is required. If you choose not to include those data, please clearly explain the reasoning for focusing only on the KinA as the partner KO.

**Journal Requirements:**

1) <carina-action-element class="ng-star-inserted" style="color: rgba(0, 0, 0, 0.87); font-family: sans-serif; font-size: 12px;">Please ensure that the CRediT author contributions listed for every co-author are completed accurately and in full.</carina-action-element> <carina-action-element class="ng-star-inserted">At this stage, the following Authors/Authors require contributions: </carina-action-element><carina-action-element class="ng-star-inserted">Ke Hu</carina-action-element><carina-action-element class="ng-star-inserted">. Please ensure that the full contributions of each author are acknowledged in the "Add/Edit/Remove Authors" section of our submission form.</carina-action-element> <carina-action-element class="ng-star-inserted">The list of CRediT author contributions may be found here: https://journals.plos.org/</carina-action-element><carina-action-element class="ng-star-inserted">plospathogens</carina-action-element><carina-action-element class="ng-star-inserted">/s/authorship#loc-author-contributions</carina-action-element>

2) We ask that a manuscript source file is provided at Revision. Please upload your manuscript file as a .doc, .docx, .rtf or .tex. If you are providing a .tex file, please upload it under the item type u2018LaTeX Source Fileu2019 and leave your .pdf version as the item type u2018Manuscriptu2019.

3) <carina-action-element class="ng-star-inserted">Please provide an Author Summary. This should appear in your manuscript between the Abstract (if applicable) and the Introduction, and should be 150-200 words long. The aim should be to make your findings accessible to a wide audience that includes both scientists and non-scientists. Sample summaries can be found on our website under Submission Guidelines:</carina-action-element>

<carina-action-element class="ng-star-inserted">https://journals.plos.org/</carina-action-element><carina-action-element class="ng-star-inserted">plospathogens</carina-action-element><carina-action-element class="ng-star-inserted">/s/submission-guidelines#loc-parts-of-a-submission</carina-action-element>

4) <carina-action-element class="ng-star-inserted">Please upload all main figures as separate Figure files in .tif or .eps format. For more information about how to convert and format your figure files please see our guidelines: </carina-action-element>

<carina-action-element class="ng-star-inserted">https://journals.plos.org/</carina-action-element><carina-action-element class="ng-star-inserted">plospathogens</carina-action-element><carina-action-element class="ng-star-inserted">/s/figures</carina-action-element>

5) We notice that your supplementary Figures are included in the manuscript file. Please remove them and upload them with the file type 'Supporting Information'. Please ensure that each Supporting Information file has a legend listed in the manuscript after the references list.

6) <carina-action-element class="ng-star-inserted">Please </carina-action-element><carina-action-element class="ng-star-inserted">amend your</carina-action-element><carina-action-element class="ng-star-inserted"> detailed Financial Disclosure statement. This is published with the article. It must therefore be completed in full sentences and contain the exact wording you wish to be published.</carina-action-element>

<carina-action-element class="ng-star-inserted">- State the initials, alongside each funding source, of each author to receive each grant. For example: "This work was supported by the National Institutes of Health (####### to AM; ###### to CJ) and the National Science Foundation (###### to AM)." </carina-action-element><carina-action-element class="ng-star-inserted">- State what role the funders took in the study. If the funders had no role in your study, please state: "The funders had no role in study design, data collection and analysis, decision to publish, or preparation of the manuscript.".</carina-action-element> <carina-action-element class="ng-star-inserted">If you did not receive any funding for this study, please simply state: u201cThe authors received no specific funding for this work.u201d</carina-action-element>

7) Figure 1: Please confirm whether you drew the images / clip-art within the figure panels by hand. If you did not draw the images, please provide (a) a link to the source of the images or icons and their license / terms of use; or (b) written permission from the copyright holder to publish the images or icons under our CC BY 4.0 license. Alternatively, you may replace the images with open source alternatives. See these open source resources you may use to replace images / clip-art:

- https://commons.wikimedia.org

- https://openclipart.org/

**Reviewers' Comments:**

Reviewer's Responses to Questions

**Part I - Summary**

Please use this section to discuss strengths/weaknesses of study, novelty/significance, general execution and scholarship.

Reviewer #1: This manuscript by Lopez et al. uses APR2, a known component of the apical polar ring (APR), as bait to isolate the APR and identify novel components. The authors identify three previously uncharacterized proteins that localize to the APR, which they designate APR9, APR10, and APR11. An APR9 knockout exhibits only a mild phenotype; however, a double mutant with kinA displays a pronounced plaquing defect that is not explained by its growth rate. The authors further link this defect to impairments in actin organization, conoid protrusion, and MIC2 secretion, contributing to defective egress. Overall, the manuscript is well written, and the data support the authors’ conclusions. I have a few points that should be straightforward to address and would strengthen the study.

Reviewer #2: This manuscript reports the identification of three novel APR proteins in Toxoplasma and the functional characterization of one of them, APR9. The authors show that depletion of APR9 has no major impact on the parasite lytic cycle. However, building on their earlier findings that APR proteins act synergistically, they generated a double KinA–APR9 mutant, which is severely impaired in parasite growth. This double mutant exhibits egress defects and reduced invasion efficiency compared to the wild type, likely as a result of combined defects affecting microneme secretion, conoid extrusion, and actin flux dynamics. The experimental data—derived from conditional knockdowns, localization studies, and functional assays are carrefully done, solid and well-controlled. While the data interpretation is logical, the section on the motility defect would benefit from further consolidation.

Reviewer #3: In this work, Lopez, Padilla, and colleagues have identified new components of the apical polar ring, which is thought to play a key role in the nucleation of the T. gondii cortical microtubules and the function of the conoid, which is essential for host cell invasion. Using immunoprecipitation with the known apical complex component APR3, three new components were identified, including APR9, which appears to be conserved in a broader range of apicomplexans than other apical ring components. Knockout of APR9 causes a modest parasite slow-growth phenotype, with occasional misplacement of the apical ring in a small portion of parasites. A dual knockout including the apical ring component kinesinA causes a very severe defect, which appears to be due to slowed parasite growth and diminished secretion of the adhesin MIC2, leading to profound entry and egress defects. Interestingly, there are only modest defects in conoid extrusion and arrangement of the cortical microtubules, even though disruption of the apical ring was thought to impact these structures. This suggests that the APC may have unexpected functions directly in secretion that are perturbed only when two components of the structure are removed.

Overall, the work in the manuscript is of high quality and well controlled. The discovery of additional components of the apical polar ring, especially one whose conservation extends out of apicomplexa, is valuable. The lack of a strong phenotype in the APR9 knockout, especially when the homolog APR4 is also knocked down, is interesting- especially as a range of other apical ring dual knockouts appear to be quite detrimental. The identification of a secretion defect in the KinesinA-APR9 dual KO, along with the paralysis and invasion/egress phenotype, is valuable information.

**********

**Part II – Major Issues: Key Experiments Required for Acceptance**

Please use this section to detail the key new experiments or modifications of existing experiments that should be absolutely required to validate study conclusions.

Generally, there should be no more than 3 such required experiments or major modifications for a "Major Revision" recommendation. If more than 3 experiments are necessary to validate the study conclusions, then you are encouraged to recommend "Reject".

Reviewer #1: Line 90. It is unclear whether APR4 or APR9 is the closest homolog to the proteins referenced in Figure 3. While this does not alter the main conclusions, inclusion of a phylogenetic tree encompassing all relevant homologs would clarify the relationship between APR4, APR9, and related proteins. This would help establish which T. gondii APR protein is most similar to those identified in other apicomplexans.

Line 189 / Figure 5D–F. While the authors likely distinguish apical and basal ends readily, this distinction is not obvious to the reader. In particular, the WT and double mutant images are difficult to interpret based on cell shape alone. A supplemental movie showing actin accumulation and directionality of movement would greatly improve clarity and should be feasible to provide.

Figure 6E. Given the reduced secretion observed in the mutant, are there any differences in MIC2 labeling between WT and the double mutant following A23187 treatment? This analysis could be informative.

Reviewer #2: Major point :

1. The motility defect of the double mutant is not entirely clear to me. The authors assessed motility by videomicroscopy after A23187 induction. Based on Figure 5B and Video S1, it is unclear whether the mutant exhibits sporadic movement or active motility within the vacuole. For the APR2 mutant, although parasites egress normally from the vacuole, no gliding motility is visible post-egress, in contrast to wild-type parasites which exhibit clear gliding motion in Video S1. Performing a classical gliding motility assay (e.g., detection of SAG1 trails) in both the APR2 and APR9/KinA mutants, along with assessing parasite dispersion after egress by IFA, would further strengthen the authors’ conclusions. This point may warrant attention, as some of the authors’ conclusions (lines 193-196 ; 218-219 ; 278-280 and 284-286) rely on the assumption that the APR2 parasite is not defective in gliding motility. However, a previous study reported that the APR2 mutant displays normal egress upon BIPPO-induced stimulation but shows impaired post-egress dispersion, consistent with a motility defect, and also produces fewer trails (Ren et al. PNAS 2024). If APR2 is truly motile under these conditions, this would challenge the current model linking conoid extrusion and actin flux dynamics to parasite motility.

Reviewer #3: My main criticism of the manuscript in its current form is why the KinesinA/APR9 dual knockout, among all the other potential dual-KOs, was selected for analysis. Excluding APR4/APR9 due a lack of a strong phenotype is reasonable, but I'm curious about what other duals were generated, and what rationale was used to select the KinesinA dual KO out of the other possibilities. Specifically, it seems that APR2 would make good sense as second knockout in conjunction with APR9, considering that it was the bait in the initial IP. While APR9 localization does not appear to change in the APR2 knockout, there seems to be some connection between the two proteins that merits further study. For example, a stronger impact on conoid extrusion and motility in the APR2/APR9 dual knockout could suggest that they are working along the same pathways. More background on APR2 in the introduction and the discussion would also be useful.

**********

**Part III – Minor Issues: Editorial and Data Presentation Modifications**

Please use this section for editorial suggestions as well as relatively minor modifications of existing data that would enhance clarity.

Reviewer #1: Line 50. In addition to the authors’ prior work (ref. 26), a recent study (Engelberg, 2025, mBio) demonstrates that γ-tubulin transiently associates with the APR to nucleate microtubules. This finding further supports the authors’ assertion regarding the APR’s role in organizing cortical microtubules and should be cited.

Line 78. The authors state that three new candidates were identified. While APR9–11 are clearly the most abundant uncharacterized proteins in the GFP-trap experiment (Table S1), several additional uncharacterized proteins (e.g., TGGT1_218720, TGGT1_275670, TGGT1_271880, TGGT1_229920) appear at levels comparable to known APR components such as APR1, APR5, and APR7. Were these proteins tested for APR localization and excluded, or were only the top hits prioritized? Clarification would be helpful.

Line 110. “mE” is defined only on line 112 but could be introduced earlier (e.g., line 81) when mEmerald knock-ins are first mentioned.

Lines 130 and 263. The manuscript refers to broad conservation of APR9; however, the relationship between APR4 and APR9 homologs remains unresolved. It may be more accurate to refer to APR4/9 when discussing conservation, as done on line 93.

Figure 2. The ordering of images in panel A makes the figure harder to follow. It would be more intuitive to present APR9 (223790) between APR11 and APR10, or simply in numerical order (APR9, APR10, APR11), consistent with Figure 1C. In panel B, the first lane is not labeled; when printed, it appears as a single band (~1.5 kb), making it difficult to interpret. It is only clear upon closer inspection that this is a ladder. Labeling the lane explicitly and/or adjusting contrast or cropping the gel to emphasize marker labels would improve clarity and consistency between the two gels.

Figure 6B. Three images are labeled as “retracted conoid” for the double mutant. It appears the authors intended to illustrate a range of phenotypes; however, panel 6D introduces a “partially extended” category that is not clearly represented in 6B. If any of the images correspond to this intermediate phenotype, they should be labeled accordingly.

Line 269. Is the P. berghei ortholog specific to APR9, or does it represent an APR4/9 homolog? Clarification would be useful.

Reviewer #2: Minors :

2. One intriguing observation is the effect of APR9/KinA depletion on MIC2 secretion, which cannot be attributed to SPMT disorganization, as these structures appear largely normal. It would be helpful if the authors could at least speculate on how microneme release might be compromised in this mutant.

3. The schematic in Figure 1A shows two PCR rings, whereas recent data indicate the presence of three.

4. In the introduction, line 50: “This observation supports the hypothesis that the apical polar ring initiates and templates new MT arrays…” A recent study (Li et al., Nature Communications, 2025) showed that in the absence of APR, SPMTs are initiated but not organized. This supports the view that the APR is not required for SPMT initiation but rather for their proper organization. Please revise the sentence accordingly.

5. In Figure 4G, the p-values of 0.01 and 0.04 are not clearly assigned. I assume they correspond to complemented clones 1 and 2.

6. A graph showing the invasion defect and standard deviation would be easier to read than a table.

7. Figure 5C: The p-values are missing from the graph.

Reviewer #3: I would recommend using vision-deficiency-compatible channels for the light microscopy images, such as magenta-green.

Fig 5, Fig6- I think the graphs are already quite small in a full-page layout and would not work well in a conventional figure layout. The graph text is already difficult to read.

Fig 6- the insets in A could be a lot clearer, it's hard to understand what you are looking at. I would perhaps inset the microtubule issue shown with the arrow as well as the apical connection issue, and make them both significantly larger.

**********

PLOS authors have the option to publish the peer review history of their article (what does this mean?). If published, this will include your full peer review and any attached files.

If you choose “no”, your identity will remain anonymous but your review may still be made public.

**Do you want your identity to be public for this peer review?**  For information about this choice, including consent withdrawal, please see our Privacy Policy.

Reviewer #1: No

Reviewer #2: No

Reviewer #3: No

[NOTE: If reviewer comments were submitted as an attachment file, they will be attached to this email and accessible via the submission site. Please log into your account, locate the manuscript record, and check for the action link "View Attachments". If this link does not appear, there are no attachment files.]

**Figure resubmission:**

While revising your submission, we strongly recommend that you use PLOS’s NAAS tool (https://ngplosjournals.pagemajik.ai/artanalysis) to test your figure files. NAAS can convert your figure files to the TIFF file type and meet basic requirements (such as print size, resolution), or provide you with a report on issues that do not meet our requirements and that NAAS cannot fix.

After uploading your figures to PLOS’s NAAS tool - https://ngplosjournals.pagemajik.ai/artanalysis, NAAS will process the files provided and display the results in the "Uploaded Files" section of the page as the processing is complete. If the uploaded figures meet our requirements (or NAAS is able to fix the files to meet our requirements), the figure will be marked as "fixed" above. If NAAS is unable to fix the files, a red "failed" label will appear above. When NAAS has confirmed that the figure files meet our requirements, please download the file via the download option, and include these NAAS processed figure files when submitting your revised manuscript.

**Reproducibility:**

To enhance the reproducibility of your results, we recommend that authors of applicable studies deposit laboratory protocols in protocols.io, where a protocol can be assigned its own identifier (DOI) such that it can be cited independently in the future. Additionally, PLOS ONE offers an option to publish peer-reviewed clinical study protocols. Read more information on sharing protocols at https://plos.org/protocols?utm_medium=editorial-email&utm_source=authorletters&utm_campaign=protocols

# Author comment: Author response to Decision Letter 1

**DOI:** 10.1371/journal.ppat.1014378.r002

21 May 2026

Attachment Submitted filename: Response_to_reviewers_RV1.pdf

# Peer review: Decision Letter 1

**DOI:** 10.1371/journal.ppat.1014378.r003

**Authors:** Margaret Phillips, Michael Reese, Margaret Phillips, Michael Reese

12 Jun 2026

Dear Dr. Hu,

We are pleased to inform you that your manuscript 'The human parasite, Toxoplasma gondii, is paralyzed without two components of the apical polar ring' has been provisionally accepted for publication in PLOS Pathogens.

Before your manuscript can be formally accepted you will need to complete some formatting changes, which you will receive in a follow up email. A member of our team will be in touch with a set of requests.

Please note that your manuscript will not be scheduled for publication until you have made the required changes, so a swift response is appreciated.

IMPORTANT: The editorial review process is now complete. PLOS will only permit corrections to spelling, formatting or significant scientific errors from this point onwards. Requests for major changes, or any which affect the scientific understanding of your work, will cause delays to the publication date of your manuscript.

Should you, your institution's press office or the journal office choose to press release your paper, you will automatically be opted out of early publication. We ask that you notify us now if you or your institution is planning to press release the article. All press must be co-ordinated with PLOS.

Thank you again for supporting Open Access publishing; we are looking forward to publishing your work in PLOS Pathogens.

Best regards,

Michael L Reese, PhD

Academic Editor

PLOS Pathogens

Margaret Phillips

Section Editor

PLOS Pathogens

Sumita Bhaduri-McIntosh

Editor-in-Chief

PLOS Pathogens

orcid.org/0000-0003-2946-9497

Michael Malim

Editor-in-Chief

PLOS Pathogens

orcid.org/0000-0002-7699-2064

***********************************************************

As you will see, the reviewers all agree that the manuscript is ready for publication in PLoS Pathogens.

Reviewer Comments (if any, and for reference):

Reviewer's Responses to Questions

**Part I - Summary**

Please use this section to discuss strengths/weaknesses of study, novelty/significance, general execution and scholarship.

Reviewer #1: It is my view that the authors have addressed all of my concerns and the manuscript and figures are improved.

Reviewer #2: The authors have satisfactorily addressed my concerns. The comparison of motility phenotypes between the mutants is now better documented, and the data support a partial motility defect in APR2.

Reviewer #3: The authors addressed all of my concerns.

**********

**Part II – Major Issues: Key Experiments Required for Acceptance**

Please use this section to detail the key new experiments or modifications of existing experiments that should be absolutely required to validate study conclusions.

Generally, there should be no more than 3 such required experiments or major modifications for a "Major Revision" recommendation. If more than 3 experiments are necessary to validate the study conclusions, then you are encouraged to recommend "Reject".

Reviewer #1: None.

Reviewer #2: (No Response)

Reviewer #3: (No Response)

**********

**Part III – Minor Issues: Editorial and Data Presentation Modifications**

Please use this section for editorial suggestions as well as relatively minor modifications of existing data that would enhance clarity.

Reviewer #1: Line 11 insert “the” after paralyzes

Reviewer #2: (No Response)

Reviewer #3: (No Response)

**********

PLOS authors have the option to publish the peer review history of their article (what does this mean?). If published, this will include your full peer review and any attached files.

If you choose “no”, your identity will remain anonymous but your review may still be made public.

**Do you want your identity to be public for this peer review?**  For information about this choice, including consent withdrawal, please see our Privacy Policy.

Reviewer #1: No

Reviewer #2: No

Reviewer #3: No

# Editor report: Acceptance letter

**DOI:** 10.1371/journal.ppat.1014378.r004

**Authors:** Margaret Phillips, Michael Reese, Margaret Phillips, Michael Reese

Dear Dr. Hu,

We are delighted to inform you that your manuscript, "

The human parasite, Toxoplasma gondii, is paralyzed without two components of the apical polar ring," has been formally accepted for publication in PLOS Pathogens.

We have now passed your article onto the PLOS Production Department who will complete the rest of the pre-publication process. All authors will receive a confirmation email upon publication.

The corresponding author will soon be receiving a typeset proof for review, to ensure errors have not been introduced during production. Please review the PDF proof of your manuscript carefully, as this is the last chance to correct any scientific or type-setting errors. Please note that major changes, or those which affect the scientific understanding of the work, will likely cause delays to the publication date of your manuscript. Note: Proofs for Front Matter articles (Pearls, Reviews, Opinions, etc...) are generated on a different schedule and may not be made available as quickly.

Soon after your final files are uploaded, the early version of your manuscript, if you opted to have an early version of your article, will be published online. The date of the early version will be your article's publication date. The final article will be published to the same URL, and all versions of the paper will be accessible to readers.

For Research Articles, you will receive an invoice from PLOS for your publication fee after your manuscript has reached the completed accept phase. If you receive an email requesting payment before acceptance or for any other service, this may be a phishing scheme. Learn how to identify phishing emails and protect your accounts at https://explore.plos.org/phishing.

Thank you again for supporting open-access publishing; we are looking forward to publishing your work in PLOS Pathogens.

Best regards,

Sumita Bhaduri-McIntosh

Editor-in-Chief

PLOS Pathogens

orcid.org/0000-0003-2946-9497

Michael Malim

Editor-in-Chief

PLOS Pathogens

orcid.org/0000-0002-7699-2064
